# Early intervention in psoriasis: Where do we go from here?

**DOI:** 10.3389/fmed.2022.1027347

**Published:** 2022-12-01

**Authors:** Paulo Antônio Oldani Felix, Ana Luisa Sampaio, Bruno Leonardo Silva, Analia Luiza Porto Viana

**Affiliations:** ^1^Dermatology Department, Hospital Federal dos Servidores do Estado, Rio de Janeiro, Brazil; ^2^Dermatology Department, Hospital Universitário Pedro Ernesto, Rio de Janeiro State University, Rio de Janeiro, Brazil; ^3^AbbVie Brazil Medical Department, São Paulo, Brazil

**Keywords:** systemic treatment, methotrexate, psoriasis, early intervention, risk stratification, therapeutic success

## Abstract

Patients with psoriasis often have comorbidities and are at increased risk of developing several complications compared with the general population. Knowledge on the role of immune mediators and systemic inflammation in psoriasis has led to the hypothesis that early intervention with systemic therapy has the potential to modify the course of the disease and reduce the risk of long-term adverse outcomes. In this article, we address some potential issues that need to be considered before early intervention can be implemented routinely. The first is determining what constitutes “early” intervention for psoriasis. A second point is whether the intervention should be considered for patients with early disease or for selected subsets based on risk stratification. A third important consideration is defining success for early intervention. Finally, adoption of early and effective intervention should be based on high-level evidence. Ideally, randomized trials would be the best strategy to compare early vs. late systemic treatment in patients with psoriasis, probably using the frequency of long-term outcomes as primary endpoint, with cutaneous and pharmacoeconomic outcomes assessed secondarily.

## Introduction

Psoriasis affects approximately 3% of adults across multiple countries, a prevalence that corresponded to approximately 7 million people in the United States in 2019 (1). Although there is geographic and ethnic variation in the prevalence of the disease, an estimate of 29.5 million adults were diagnosed with psoriasis worldwide in 2017 (2). The disease is heterogeneous both in its cutaneous manifestations and in terms of the associated comorbidities. Psoriatic arthritis, for example, develops in up to 30% of patients with psoriasis at an estimated annual rate of 2.7% (3, 4). Patients with psoriasis are also at increased risk of developing cardiovascular complications, diabetes mellitus, obesity, inflammatory bowel disease, and nonalcoholic fatty liver disease compared with the general population (5, 6). In addition to these comorbidities, positive associations have been noted between psoriasis and mental disorders, stroke, lymphomas, and non-melanoma skin cancer (7). Given the chronic, recalcitrant, and disabling nature of psoriasis, the World Health Organization (WHO) has considered it a serious non-communicable disease that adversely and sometimes needlessly affects many people owing to incorrect or delayed diagnosis or inadequate treatment (8).

Knowledge about the role of immune mediators and systemic inflammation in psoriasis has considerably evolved over the past two decades, leading to the introduction of biological agents and the hypothesis that early intervention with systemic therapy has the potential to modify the course of the disease and reduce the risk of long-term adverse outcomes, such as psoriatic arthritis and cardiometabolic disorders (5, 9, 10). However, unlike disorders such as rheumatoid arthritis, psoriatic arthritis, and Crohn's disease, in which permanent structural damage can occur, in plaque psoriasis it may be more appropriate to adopt the concept of cumulative life-course impairment (CLCI) to assess long-term therapy intervention benefits. This concept has been proposed to indicate the cumulative, life-long effects of physical and psychological factors, as well as the economic and social consequences, of psoriasis in patients' lives (11). Increased understanding and acceptance of CLCI may help to identify patients at risk and critical periods for optimizing interventions in psoriasis (11, 12). Several issues need to be addressed before early intervention can be considered standard of care. In this article, we review relevant topics to consider regarding the potential role of early intervention with systemic agents in plaque psoriasis.

## Conceptualizing early intervention

As in other medical fields, early intervention is an appealing concept in psoriasis and not a new idea (9). In discussing this concept, we restrict our focus to patients with skin involvement who do not present with and who are not candidates for intervention based on current guidelines and drugs in approved indications for psoriatic arthritis (13, 14). Because psoriatic arthritis is preceded by skin involvement in nearly 90% of patients and by an average of 7 years, early identification of arthritis, enthesitis, or dactylitis by dermatologists remains paramount (3, 15, 16). Of note, the important role of early and effective treatment in patients with psoriatic arthritis is well established (15–18). Likewise, the equally important topic of under treatment in psoriasis will not be addressed, but we note that it still requires greater attention from physicians, medical societies, and funding agencies, given that up to 50% of patients with mild psoriasis, 35% of those with moderate psoriasis, and 30% of those with severe psoriasis are untreated (19, 20).

The first point to consider in exploring the worth of early intervention is how to define it. At present, there seems to be no universally accepted definition. In principle, one approach could be the measurement of the time to intervention from different stages of the disease. For example, time since diagnosis is an obvious candidate, but no clear cutoff currently exists (9). In recent pivotal trials of biologicals, there is an average lapse of 15–20 years between symptom onset and treatment of psoriasis, in most cases with prior different therapies. In the specific context of psoriatic arthritis, for which early systemic treatment is recommended, early-stage disease often denotes the first 2 years from symptom onset (21, 22), whereas a cutoff of 5 years has been used to distinguish between early and late treatment with biologicals (23). On the other hand, intervention in psoriatic arthritis is recommended as soon as the diagnosis is made, and a delay in diagnosis by more than 6 months has been associated with worse outcomes (24).

In patients with plaque psoriasis, the unpredictable course of the disease and development of complications make it challenging to base a definition of early intervention solely on time since diagnosis. In that regard, it will be important to establish whether prior topical therapy or phototherapy, when indicated, are a prerequisite for the definition of early intervention with systemic agents. The same reasoning can be applied when considering early change from conventional therapies to biologicals (25). Age of onset of psoriasis might also be relevant in proposing a definition for early intervention, given its association with complications, as discussed ahead. Thus, the scientific community will need to agree on at least a working definition going forward and to propose studies that can both validate such a concept and test the utility of applying it in patient treatment. We believe that a provisional definition should consider early intervention as the use of systemic agents in patients with mild or moderate skin involvement who would not otherwise be candidates for phototherapy and systemic treatments based on current guidelines and their approved indications.

## Risk stratification

A second important point to discuss is whether early intervention should be considered for all patients with mild or moderate psoriasis or for selected subsets based on an increased risk. Risk stratification could be based on several factors, such as age, presence of comorbidities, disease severity, genetic profile, or other factors associated with likelihood of complications from psoriasis, including psoriatic arthritis (15). Ideally, risk stratification should be based on validated prognostic factors and models. Notwithstanding the following discussion on candidate prognostic factors for risk stratification, validated models are not available for predicting the progression of psoriasis to more aggressive forms or the onset of arthritis or other complications, perhaps because of the heterogeneity of this disease. Moreover, the ascertainment of prognostic factors for development of complications is often made difficult by issues related to study design and the need to differentiate association from causation (26). In the attempt to make that differentiation, confounding is a constant threat unless dealt with appropriately. This can be illustrated by the finding that the use of conventional systemic therapies was associated with increased mortality in a large observational study of patients with psoriasis; because patients with psoriasis have increased mortality compared with the general population, the use of conventional systemic therapies is probably a marker of disease severity (27). In some cases, it may be very difficult to assess the directionality of a given association, and therefore to define if a certain condition (e.g., metabolic syndrome) should be considered a risk factor for future complications or one of the complications to be prevented. Immunosuppressive treatment can arguably be considered a confounding factor in the causation of serious infections, which are more frequent among patients with vs. without psoriasis (28). Finally, some conditions can be either a complication of the disease or of its treatment, something illustrated by concerns about cardiovascular events among patients treated with certain biologicals (29).

### Age of onset

There is conflicting evidence in the literature regarding the prognostic role of age of onset. In adults, the relationship between age of onset of psoriasis and the development of arthritis, for example, is not clear (30, 31). Likewise, the interplay between age of onset and development of cardiovascular complications in psoriasis is complex and appears to be mediated by disease severity. A nationwide cohort study from Denmark found that the risk of a first myocardial infarction was increased only in patients aged <50 years with severe psoriasis (32). A retrospective cohort study from Canada found that patients with onset of psoriasis younger than 25 years were more likely to have a myocardial infarction than patients with later onset (33). On the other hand, using age of onset as a criterion for risk stratification may need to take into account the finding from an observational study that early-onset psoriasis (i.e., ≤40 years of age) is less likely to respond to systemic therapy than late-onset disease (34). Whether this is due to increasing resistance to therapy resulting from prolonged disease activity remains speculative. Once again, we caution about the limitations of observational studies (30). Thus, future studies to define and validate the role of early intervention in psoriasis will be important to stratify patients or conduct subgroup analyses based on age of onset.

### Severity and phenotype of psoriasis

With regard to the severity of plaque psoriasis, which accounts for nearly 90% of all cases, the disease is typically mild and can be managed with topical treatment alone (35). Nonetheless, there can be considerable functional impairment from psoriasis even if most patients will have no irreversible or progressive skin damage (9, 35). The effect of the disease on quality of life is related to the severity and duration of active psoriasis, the extent and location of lesions, the presence of associated arthritis, the frequency of relapses, and the need for treatment (9). Additionally, more severe psoriasis is a risk factor for psoriatic arthritis, which can plausibly be triggered by a higher burden of skin inflammation (5, 15, 26). Because patients presenting with more aggressive disease or with psoriatic arthritis are often treated with systemic agents, and given that biologicals are more effective than conventional systemic treatment in moderate to severe plaque psoriasis in terms of clearance of skin lesions over a 24-week period (36), the question naturally arises whether these agents should be used earlier in the disease course. Disease phenotype and nail, scalp, and inverse psoriasis are associated with an increased risk of arthritis (3, 15). Therefore, the role of disease severity and phenotype in determining the appropriateness of early intervention requires elucidation.

Dose tapering could serve as a potential model to attest the benefits of early intervention in modifying the course of psoriasis and psoriatic arthritis. Biologic tapering seems to be effective and safe in psoriasis patients with stable low disease activity or clinical remission, but consistent evidence is lacking (37). Atalay et al. (38) investigated clinical predictors for successful dose tapering, but no variables showed a predictive value, including disease duration, age of onset and age at inclusion. Once again, future studies will benefit from stratifying or analyzing subgroups of patients based on prognostic or predictive features.

### Metabolic risk factors

Metabolic risk factors are also candidates in the attempt to stratify risk because patients with psoriasis are at increased risk for major adverse cardiovascular events (5, 6, 39). The association between psoriasis and these complications is supported by different lines of evidence. Studies have shown that patients with psoriasis have a high burden of subclinical atherosclerosis (6), whereas others have shown an increased frequency of major adverse cardiovascular events compared to controls (40–42). However, conflicting results have been reported in some large-scale studies assessing the risk after adjustment for known risk factors for cardiovascular events. In one study from the United Kingdom, neither psoriasis nor severe psoriasis was associated with the risk of major cardiovascular events in the short term (3–5 years) after adjusting for known risk factors (43). In a second UK study, the association was present, but appeared to be mediated by systemic treatment, because it was only significant among patients not receiving these drugs (44). Likewise, a prospective study from the Netherlands with a mean of 11 years of follow-up found no increase in risk of cardiovascular morbidity for patients with mostly mild psoriasis (45). Moreover, results from observational studies on the positive association between psoriasis and cardiovascular disease have not been confirmed by a randomized controlled trial (RCT) assessing the role of anti-tumor necrosis factor-alpha therapy in reducing vascular inflammation (46). Whether the conflicting results of these studies on the association between psoriasis and cardiovascular events are due to methodologic issues, such as study design or analytical method, remains uncertain. Once obesity, diabetes, and metabolic syndrome are associated with cardiovascular disease [also in patients with psoriatic arthritis (47, 48)], it remains to be determined whether the association between psoriasis and cardiovascular disease, if causal, is due to systemic inflammation, traditional risk factors, or genetic factors (9). Observational studies suggest that obesity is a risk factor for the development of psoriatic arthritis among patients with psoriasis, and that its control can reduce this risk (48–50). Once again, there is an interplay of different factors because psoriatic arthritis is also associated with diabetes (48, 51). The role of metabolic risk factors as an aid to risk stratification remains to be determined.

### Genetic factors

Despite the well-known contribution of genetic factors to the pathogenesis of psoriasis (9, 39), most of the knowledge on the association between such factors and disease severity or progression relates to psoriatic arthritis (15, 52). It is well known, for example, that first-degree family history of psoriatic arthritis in patients with psoriasis confers an increased risk of psoriatic arthritis (15). Likewise, several human leukocyte antigen variants, single nucleotide polymorphisms, and other genetic variants have been identified that bear implication on the pathogenesis of psoriatic arthritis (52). It is therefore conceivable that risk stratification for early intervention could incorporate genotypic information, especially in light of increasing emphasis on collection and analysis of samples for translational research related to patient diagnosis and management in psoriasis and other diseases. Moreover, genetic markers could serve as predictive factors for response to specific targeted therapies, further strengthening their role in early treatment decisions (53, 54). In that regard, correlative analysis of completed studies with those therapies remain an essential step toward the goal of providing precision medicine in psoriasis.

## Assessing the benefit of early intervention

A third important consideration is the definition of success for early intervention; without such definition, the value of early treatment cannot be ascertained. In that regard, psoriatic arthritis provides an interesting illustration because the definition of minimal disease activity as a valid treatment target allowed implementation of RCTs using it as an efficacy endpoint among patients with early-stage disease (22). Even though the concept of minimal disease activity is useful, the fact that early intervention aims at controlling skin disease as well as preventing complications means that long-term results would be needed to validate early success as a potential surrogate for long-term outcomes. Unfortunately, such outcomes are not usually collected in RCTs of plaque psoriasis, and most of the information on these outcomes originates from observational studies, usually retrospective. However, there is ample rational basis for considering that early control of inflammatory activity will result in long-term benefit for patients, as already noted (5, 9, 15). Ideally, early intervention should have an overall effect on CLCI, and this should also be tested going forward.

### Conventional treatment targets

Systematic work has been done in the attempt to propose treatment targets in plaque psoriasis (Table 1) (29, 55–63). These initiatives explored the concept of treat-to-target, used successfully in rheumatoid arthritis and arguably in psoriatic arthritis. Some authors have suggested that a possible target in psoriasis could be an absolute Psoriasis Area and Severity Index (PASI) ≤1 and involvement of ≤3% of the body surface area (9). It should be noted, however, that the tools summarized in Table 1 have been developed mostly for clinical trials, rather than clinical practice. The Canadian initiative, for example, does not consider PASI, often the basis for the primary or a key secondary endpoint in clinical trials (56). Also, the tools differ in terms of being unidimensional or multidimensional, according to whether aspects related to quality of life, patient satisfaction, and treatment safety are also considered in the assessment. These targets refer to skin disease, and whether their achievement is associated with improved long-term outcomes and decreased frequency of complications from psoriasis remains to be determined. Interestingly, inspection of Table 1 suggests a temporal trend for the use of more stringent PASI criteria (from 75 to 90% improvement [PASI 75 and PASI 90, respectively]), thus reflecting the advent and efficacy of biologicals (64, 65). More recently, studies have used complete resolution of lesions (PASI 100) as an endpoint that has been met more frequently with biologicals than with conventional therapies in moderate to severe psoriasis (66). These findings suggest that PASI 100 could be a feasible target for early intervention for psoriasis.

**Table 1:** Published proposed targets in psoriasis.

**Group (year of publication)**	**Treatment target related to the skin**	**Other domains considered in response assessment**
European consensus (2011) (55)	A reduction in PASI ≥75%	DLQI ≤5 used as an aid in cases of intermediate responses
Canadian expert opinion paper (2015) (56)	PGA 0	None
Spanish Academy of Dermatology and Venereology (2016) (57)	A reduction in PASI ≥90% and PGA ≤1 or minimal and controllable localized involvement with topical treatments (PGA ≤2 and PASI <5)	DLQI ≤1
National Psoriasis Foundation (2017) (58)	Involvement of ≤1% of BSA in the first 3 months and during maintenance	None
French Society of Dermatology (2019) (59)	PASI ≥90 and absolute PASI ≤3 or PGA 0–1	DLQI 0 or 1
Belgian consensus panel (2020) (60)	A reduction in PASI ≥90% or PGA ≤1, itch VAS ≤10 mm, absence of disturbing lesions, at 12 weeks	DLQI ≤1, incapacity daily functioning VAS ≤10 mm, safety (≤ mild adverse effects), and full tolerability of treatment
British Association of Dermatology (2020) (61)	Minimal response defined as a reduction in PASI ≥50% (or as percentage of BSA if PASI is not applicable)	Clinically relevant improvement in physical, psychological, or social functioning (e.g., ≥4-point improvement in DLQI or resolution of low mood)
Japanese Dermatological Association for Psoriasis (2020) (62)	A reduction in PASI ≥90%	DLQI score of 0 or 1
Brazilian Consensus of Psoriasis 2020 (2021) (29, 63)	PASI ≥90 or absolute PASI <3 (for patients in use of biologics)	DLQI 0 or 1, PGA 0 or 1, BSA < 3

BSA, body surface area; DLQI, Dermatology Life Quality Index; PASI, Psoriasis Area and Severity Index; PGA, physician's global assessment; VAS, visual analog scale.

In summary, no universally accepted metric currently exists that could be used as either a therapeutic target in clinical practice or a preferred efficacy endpoint in clinical trials and, at the same time, serve as the overarching target of early treatment. From our assessment of the current literature, we believe this metric should be sensitive enough to capture commonly accepted success criteria related to skin involvement (Table 1) and to predict long-term outcomes and complications.

### Immunologic markers

The skin is the largest organ of the human body and contains an estimated 20 billion T cells, which are responsible for local defense against pathogens and tumors and for maintaining tolerance to self-antigens (67). When T cells are activated by antigen-presenting cells, a small fraction of them differentiate into precursor memory T cells, which may ultimately differentiate into several subsets. One of the subsets receiving increasing attention is tissue-resident memory CD4+ and CD8+ T cells (67, 68). In addition to their normal function of responding rapidly to pathogenic challenges, there is emerging evidence that tissue-resident memory T cells are involved in the recurrence of chronic inflammatory skin disorders, including psoriasis (67–69). It has been postulated that, in psoriasis, the recurrence of lesions in the same location is linked to the presence of tissue-resident memory T cells in those specific areas of the skin, even after successful clearance induced by treatment with biologicals (68, 70). High efficacy of biologicals targeting interleukin-17 and interleukin-23 pathways may be linked to effects on memory T cells (69). Also, it has been shown that tissue-resident memory CD8+ T cells derived from the skin are more prevalent in patients with psoriatic arthritis than in those with psoriasis, which suggests that disruptions in skin homeostasis contribute to arthritis development (71).

There is increasing interest in better understanding the role of regulatory T cells (Tregs), which play a fundamental role in immune homeostasis by helping to prevent autoimmune disease through the suppression of immune responses (72). In psoriasis, Treg dysfunction is associated with disease exacerbation, and some of the available treatments modulate the number and function of Tregs. For example, phototherapy and antibodies targeting interleukin-17 and interleukin-23 pathways can, at least in part, rescue the suppressive function of Tregs. The balance between Th17 cells and Tregs can be restored through several different mechanisms modulated by psoriasis therapy (73). Therefore, these and other recent results in this field suggest that quantitative and qualitative assessment of immunologic markers may lead to the development of signatures predicting response to treatment and allow for individualized approaches to achieve earlier positive outcomes.

## What is the evidence for early treatment?

Early and effective intervention can be considered the standard of care for psoriasis only if based on high-level evidence. Ideally, such evidence should come from RCTs testing the value of early intervention in preventing adverse long-term outcomes, such as psoriatic arthritis and comorbidities associated with psoriasis, as well as their effect on the CLCI. These RCTs would compare the strategy of early vs. deferred treatment with systemic conventional or biological agents in patients with mild psoriasis, probably using as primary endpoint the frequency of long-term outcomes. At present, such trials are scarce and may be difficult to design and conduct. The phase 3b trial GUIDE (NCT03818035) aims to investigate the impact of early intervention with guselkumab, an IL-23 inhibitor, on the clinical response and maintenance of response after drug withdrawal in subjects with moderate-to-severe plaque psoriasis. This study has enrolled a total of 880 subjects with short (≤2 years) or longer (>2 years) disease duration and efficacy and safety results are expected for the near future (74). Given the variable and potentially long interval between onset of psoriasis and the development of comorbidities, RCTs would have to be large and lengthy to have enough power to detect a significant effect from the intervention. Moreover, the control arm would probably receive topical therapy initially and systemic therapy in a deferred fashion, and the effects of these treatments would need to be considered. In these RCTs, conventional assessment of skin manifestations (e.g., using PASI 75, 90, or even 100) would be key secondary endpoints to ensure adequate control of psoriatic lesions. Alternatively, RCTs would have skin outcomes as primary endpoints, and would continue to collect data for several years on secondary outcomes related to complications. They could also be conducted using potential surrogate markers for long-term efficacy, such as immunologic or imaging parameters, as discussed previously; the greater frequency of such outcomes could provide RCTs with higher sensitivity and make them smaller and faster to conduct. Finally, pharmacoeconomic outcomes should be assessed in these large trials, given the direct cost of treatments and the cumulative negative financial effect of the disease over time (11).

### Evidence from other conditions

Even though evidence from prospectively designed, large RCTs is not yet available, and given the importance of this issue, some insight can be obtained by examining the literature on psoriatic arthritis and other chronic immune-mediated disorders, as well as other types of evidence originating from research on psoriasis.

Observational studies and retrospective analyses of clinical trials suggest that earlier introduction of systemic therapy is advantageous in patients with psoriatic arthritis (23, 24, 75–77), and this suggestion has been used as the basis for the design of RCTs in these patients. In the Tight Control of Inflammation in Early Psoriatic Arthritis (TICOPA) trial, a treat-to-target approach significantly improved joint outcomes at 48 weeks for newly diagnosed (<2 years) patients, in comparison with a standard approach (22). The TICOPA trial did not test directly whether early intervention is superior to deferred treatment, but the focus on more recently diagnosed patients is a step forward. Thus, although the true concept of early intervention in subclinical psoriatic arthritis remains to be tested, there is ample rational basis for it (5, 15).

The same rationale for early intervention exists for other immune-mediated chronic inflammatory diseases. Rheumatoid arthritis is the prime example of a disease for which a progressive shift to earlier treatment has led to improved results over time, with incorporation of early treatment into practice guidelines (78). Early treatment initiation (usually ≤3–6 months), risk stratification, and more aggressive therapy (e.g., combination therapy) have been typically associated with improved outcomes in the management of the disease (9, 78, 79). A similar approach is being proposed in Crohn's disease and ulcerative colitis, with evidence from observational studies and retrospective analyses of clinical trials favoring early intervention being summarized recently (9, 79, 80). The similarities between plaque psoriasis and all these conditions, and the fact that different therapeutic targets are relevant in several of them, support a common view that early intervention can be beneficial to patients.

### Current evidence in psoriasis

Even though large-scale RCTs do not seem to be on the horizon, except for the GUIDE study (74), smaller studies are under way and may shed light on the role of early intervention, at least in moderate to severe psoriasis. One such study is STEPin (NCT03020199), which aims to compare secukinumab vs. narrow-band ultraviolet B in 205 patients with new-onset disease (≤12 months) and no prior systemic treatment or phototherapy. The primary efficacy endpoint in this RCT is the proportion of patients who achieve PASI 90 at week 52 (81). Observational studies can be used to provide further support for the hypothesis under discussion here, but caution should be exercised regarding the assessment of causality. Recent retrospective studies suggest that the development of psoriatic arthritis after therapy initiation is less frequent among patients with psoriasis treated systemically than those treated topically or with phototherapy (narrowband UVB) (82, 83).

## A proposed framework for research

**Figure 1:**
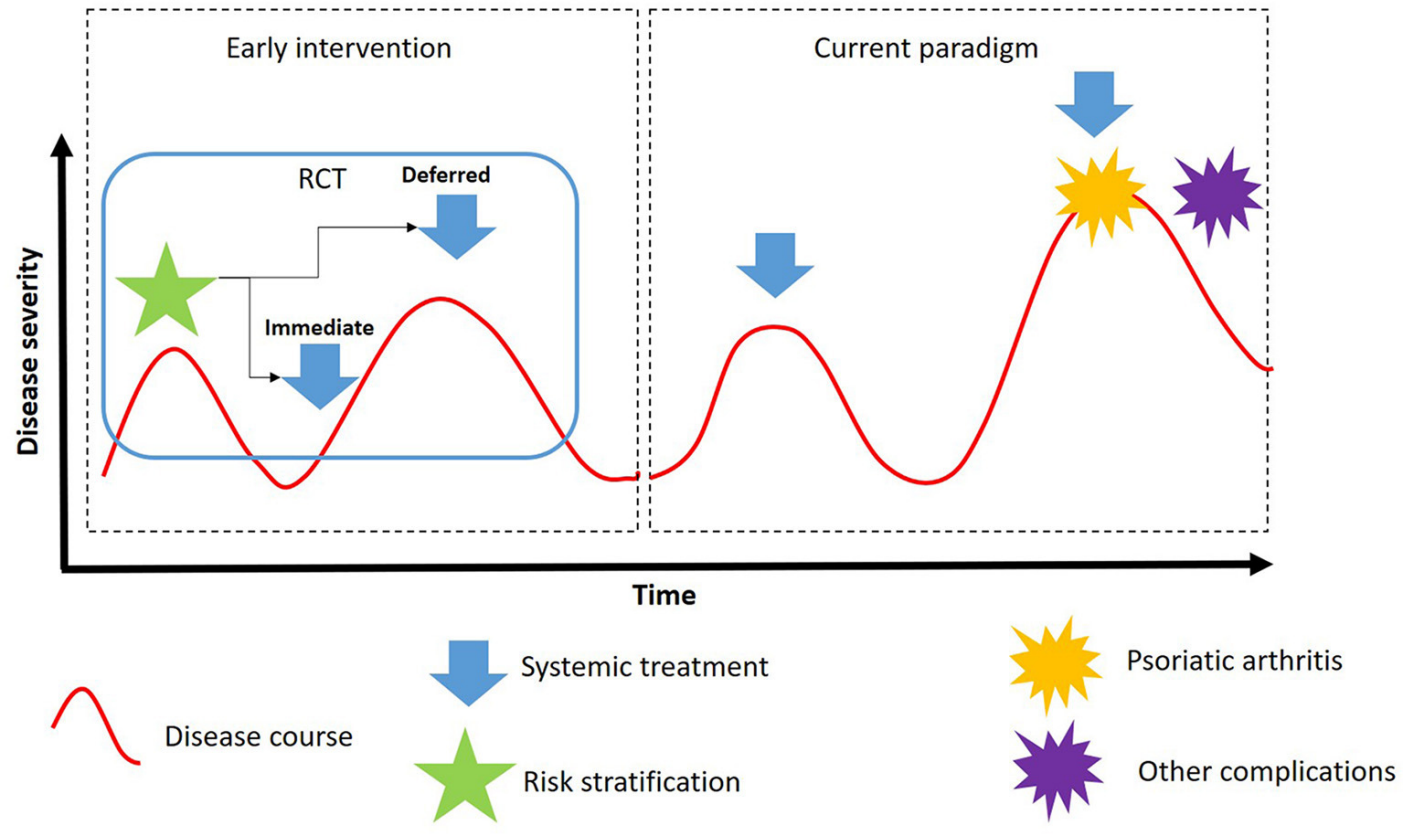
Proposed model to test the role of early intervention in plaque psoriasis (RCT, randomized controlled trial).

We propose that the required framework for validating the concept of early intervention should be able to test the validity of the model depicted in Figure 1. According to this model, plaque psoriasis has a variable course, but one that may be predicted with sufficient accuracy—with the use of validated models—to permit early intervention to ameliorate skin symptoms and prevent adverse long-term outcomes. The choice of the most suitable agents and treatment sequences for early intervention would require careful discussion (9). With some of the conventional therapies, a major limitation for long-term treatment is cumulative toxicity, such as liver toxicity from methotrexate, renal toxicity from cyclosporine, and skin carcinogenesis from phototherapy (65); thus, the merits of using a continuous vs. intermittent regimen would also need to be assessed.

## Conclusion

Thanks to recent improvements in systemic treatment for mild to moderate psoriasis, it is now possible to envision prevention of long-term adverse outcomes and earlier intervention as laudable and achievable therapeutic goals. The challenges ahead include the definition of early intervention, the creation of risk stratification tools sufficiently predictive of long-term outcomes, and the implementation of RCTs using sensitive endpoints that are ultimately associated with those outcomes and that generate the evidence base currently missing. The overall effect of early intervention on the CLCI and cost considerations should be factored into the research agenda. There is ample scientific rationale, as well as some observational evidence, that early intervention could benefit patients and be cost-effective in different healthcare scenarios. Alongside wider awareness of the disease and its treatment, and the continued development of effective and safe agents, early intervention should be a next milestone in psoriasis research.

## Author contributions

AV and BS conceived the manuscript. AV drafted the manuscript. PF, AS, and BS provided literature search and reviewed the manuscript. All authors were involved in scientific discussion of the review. All authors contributed to the article and approved the submitted version.

## Funding

AbbVie funded medical writing services provided by Dendrix (São Paulo, Brazil).

## Conflict of interest

PF has served as investigator and/or consultant to advisory boards and as paid speaker for AbbVie, Amgen, Boehring Ingelheim, Ely Lilly, Janssen, Leopharma, Novartis, Pfizer, Sandoz, Sanofi, and UCB. AS has served as consultant to advisory boards for Novartis and Janssen, and paid speaker for Novartis, Janssen, and Leopharma. AV is a former employee of AbbVie and may own AbbVie stock or stock options. BS is an employee of AbbVie and may own AbbVie stock or stock options. The authors declare that this study received funding from AbbVie. The funder participated in the interpretation of data and the review and approval of the content.

## Publisher's note

All claims expressed in this article are solely those of the authors and do not necessarily represent those of their affiliated organizations, or those of the publisher, the editors and the reviewers. Any product that may be evaluated in this article, or claim that may be made by its manufacturer, is not guaranteed or endorsed by the publisher.
